# Endostatin a Potential Biomarker for Heart Failure with Preserved Ejection Fraction

**DOI:** 10.5935/abc.20170144

**Published:** 2017-11

**Authors:** Michael Coll Barroso, Philip Boehme, Frank Kramer, Thomas Mondritzki, Till Koehler, Jan-Erik Gülker, Martin Karoff, Wilfried Dinh

**Affiliations:** 1 Klinik Königsfeld der Deutschen Rentenversicherung Westfalen in Ennepetal (NRW) - University Hospital; 2 Bayer AG - Drug Discovery - Experimental Medicine; 3 Bayer AG - Drug Discovery - Therapeutic Research Groups Cardiovascular III; 4 Department of Cardiology - HELIOS Clinic Wuppertal - University Hospital Witten/Herdecke; 5 Department of Cardiology - HELIOS Clinic Krefeld

**Keywords:** Heart Failure, Endostatins, Natriuretic Peptides, Biomarkers, Stroke Volume

## Abstract

**Background:**

Endostatin is a circulating endogenous angiogenesis inhibitor preventing neovascularization. Previous studies demonstrated the prognostic value of Endostatin among patients with heart failure with reduced ejection fraction (HFrEF). However, the role of Endostatin among patients with heart failure with preserved ejection fraction (HFpEF) remains unclear.

**Objective:**

This study aimed to investigate the association between serum Endostatin levels, natriuretic peptide levels and the severity of left ventricular diastolic dysfunction and the diagnosis of HFpEF.

**Methods:**

Endostatin serum concentrations were measured in 301 patients comprising 77 HFpEF patients, 169 patients with asymptomatic left ventricular diastolic dysfunction (ALVDD), and 55 controls with normal cardiac function.

**Results:**

Endostatin serum levels were significantly elevated in patients with HFpEF (median/interquartile range 179.0 [159-220]) and ALVDD (163.8 [145.4-191.3]) compared to controls (149.1 [130.6-176.9]), p < 0.001 and p = 0.004, respectively) and significant correlated with N-terminal pro B-type natriuretic peptide (NT-proBNP).

**Conclusions:**

This hypothesis-generating pilot study gives first evidence that Endostatin correlates with the severity of diastolic dysfunction and may become a novel biomarker for HFpEF. We hypothesize a rise in Endostatin levels may reflect inhibition of adaptive angiogenesis and adverse cardiac remodeling.

## Introduction

The patient population affected by heart failure (HF) is growing in a constant manner. This is because of an aging society, western lifestyle and improved acute clinical care (e.g. after myocardial infarction).^1^ Although, the treatment of chronic conditions improved over the last decades, mortality and morbidity rates in this patient population are amongst the highest for western healthcare systems.^2^ In the United States (US) HF is the leading cause for hospitalization for patients older > 65 years of age.^3^ In 2030 the direct costs for heart failure will reach 70 billion US$ in the US alone.^4^ Half of the patients affected by HF present with a diastolic dysfunction and a preserved ejection fraction (HFpEF), with this proportion increasing.^5^ Clinical data proves that those patients suffering from a reduced ejection fraction (HFrEF) show better outcomes compared to HFpEF patients.^6-8^ A reason might be that no therapy has been shown to improve outcomes in HFpEF.^9^ Current therapeutic options including fluid management, blood pressure control and physical exerciseto relief patients' symptoms. A major drawback regarding the development of new therapies for HFpEF, is the absence of clear diagnostic criteria.^10^ This makes the definition of patient populations for clinical studies difficult. At present, the diagnosis is solely based echocardiography. Especially, the separation between HFpEF and HFrEF is even more challenging and misleading in patients with newly diagnosed HF.^11^ Therefore new strategies for disease phenotyping in HF are urgently needed. New biomarkers may achieve better disease phenotyping.^12^ Although, many reports have been published on HF biomarkers over the last decades, the impact on clinical decision making is still limited.^13^ BNP/NTproBNP demonstrated high clinical utility to identify patients at high risk for heart failure hospitalization and death. However, in this context these markers for clinical studies are only applicable in relatively stable patients and not in terminal HF patients. Furthermore, the use of BNP/NTproBNP in clinical practice to optimize therapy with drugs, which are known to improve patient`s outcome is suitable.^14^ However, BNP/NTproBNP is not accepted as surrogate endpoint and can only exploratorily be used as endpoint in clinical trials. The appraisal of clinical utility of BNP/NTproBNP manifests in the current guidelines for the management of heart failure.^15^ A number of publications propose Endostatin, a potent angiogenesis inhibitor, known mostly from oncology, as a potential new HF biomarker candidate.^16-18^ Most importantly Gouya et al. reported in a prospective observational cohort study in 151 HF patients, a correlation between elevated circulating Endostatin levels and mortality. Furthermore, this study showed a clear association between Endostatin levels and progressing diastolic dysfunction, the key characteristic of HFpEF.^19^ This is why we hypothesize that Endostatin could potentially be a biomarker suitable to diagnose and disease phenotype HFpEF patients. In the present study, we aimed to investigate the sole role of Endostatin as a biomarker for HFpEF and diastolic dysfunction.

## Methods

The study protocol was approved by the Ethics Committee of the Private University of Witten/Herdecke, Germany (project n°. 91/08) and conducted in accordance with the Declaration of Helsinki. Signed written informed consent was obtained from all patients.

### Study population

Participants of the prospective observational cohort study were patients contacting the HELIOS Klinikum Wuppertal Heart Center (Wuppertal, Germany) for elective coronary angiography or diagnostic work-up of heart failure. Patients with a stable or suspected coronary artery disease (CAD) and/or a diagnostic workup of CHF were included in the study. The exclusion criteria were: left ventricular ejection fraction (EF) < 50%, known CAD with progressive chest pain within the last month, coronary angioplasty or myocardial infarction within 6 weeks, hypertrophic cardiomyopathy, moderate-to-severe valvular heart disease, uncontrolled hypertension, atrial fibrillation or other severe arrhythmias, serum-creatinine > 2,0 mg/dl. Patients selected for the control group had to have no history or symptoms of CHF, a normal ejection fraction > 55%, a ratio of the early diastolic transmitral velocity (E) and the early diastolic tissue Doppler velocity (E´) of < 8, and normal NTproBNP values. A total of 301 patients were recruited and assigned to three groups based on echocardiographic diagnostic criteria as recommended by the European Society of Cardiology.^20^ The control group consisted of 55 patients (29 males) with normal diastolic function (DF). The group with asymptomatic left ventricular diastolic dysfunction (ALVDD) contained 169 patients (95 males) with E medial < 8 cm/s, E/E' medial ratio 8-15 and NT-proBNP levels < 220 pg/ml. The group with HFpEF comprised 77 patients (46 females, 31 males) displaying ALVDD Grad II - III with E/E´ratio > 15, NT-proBNP levels > 220 pg/mL and current or previous signs or symptoms of heart failure.

### Echocardiography

Echocardiography was performed using a standard ultrasound system (Vivid 7, General Electric, Milwaukee, Wisconsin). A complete transthoracic study was performed including 2D, M-mode, spectral and colour Doppler techniques following current recommendations and guidelines.^21,22^ The left atrium volume index (LAVI) was calculated using the biplane area-length method. Left ventricular EF was measured by means of the modified biplane Simpson's method.^23^ Left ventricular mass index (LVMi) was computed with the Devereux formula indexed to the body surface.^22^ HFpEF was defined in accordance with the EAE/ASE recommendation, based on the assessment of left ventricular diastolic function.^24^ Primary measurements included mitral inflow peak early (E-wave) and late (A-wave) diastolic filling velocities as well as systolic (S) and early diastolic (E') mitral annular velocities whereat in each case three consecutive beats were measured and averaged. Conventional transmitral flow was measured with Pulse-waved Doppler (PW). PW tissue Doppler imaging (DTI) was performed at the junction of the septal and lateral mitral annulus in the apical 4-chamber view. Based on primary measurements E/A and E/E' ratios were calculated.

### Laboratory analysis

Peripheral venous whole blood samples were taken after 5 minutes at rest for routine laboratory testing (OGTT, total cholesterol, LDL cholesterol, HDL cholesterol, triglyceride, creatine, leucocytes, hemoglobin, creatin kinase, TSH, hsCRP, GOT, GPT). Blood was drawn into pyrogen-free tubes without any additives, centrifuged at room temperature, aliquoted and stored at -80°C. All laboratory analysis were outsourced to Roche Diagnostics (Penzberg, Germany) and performed on blinded samples. For analysis of plasma NT-proBNP the Elecsys 2010 NT-proBNP assay (Roche Diagnostics, Mannheim, Germany) was used. For measurement of Endostatin the ELISA assay of R&D Systems (Minneapolis, MN USA) was used. All assays were performed according to manufacturer's recommendations.

### Statistical analysis

All analyses were performed using SPSS statistical software (SPSS 19.0, Chicago, IL, USA). The data are presented as median with 25^th^/75^th^ percentiles (interquartile range) for continuous variables or as absolute numbers and corresponding percentages for categorical variables unless otherwise specified. Log transformed values were used for analysis as appropriate. A p-value < 0.05 was considered statistically significant. We used the Kolmogorov-Smirnov test as appropriate to test for normal distribution. The Mann-Whitney U-test was used to analyze differences between the medians of two groups and the Kruskal-Wallis test to test the equality of medians among more than two distinct groups. Fisher's Test was used for the comparison of two sets of binary variables and the χ^2^ test to evaluate differences in proportions in more than 2 sets of categorical variables. Endostatin and NT-proBNP levels were compared across subjects with normal diastolic function, mild ALVDD and HFpEF by the Mann-Whitney U-test, and Jonckheere-Terpstra test. Spearman rank correlation was used to identify variables associated with Endostatin. A multivariable model was included to predict the presence of HFpEF and included the following covariates: Endostatin, age, gender, diabetes, hypertension, coronary artery disease and body mass index. Due to the exploratory nature of this study, there is no minimum required sample size.

## Results

### Study population characteristics

Table 1 provides an overview of the clinical characteristics of all 301 patients included in our study. The three groups showed comparable diastolic blood pressure, resting heart rate and history of myocardial infarction and stroke. Patients with mild ALVDD or HFpEF were older, more obese, had a higher systolic blood pressure on average and showed a higher prevalence of comorbidities including CAD and coronary artery bypass graft, as well as cardiovascular disease risk. In addition, treatments varied across groups.

**Table 1:** Baseline characteristics of the study population. Values are median (25 75interquartile range) or absolute numbers and percentage (%)

Clinical variables(median/interquartile range or %)	Studied patient groups			p value
	Control (n = 55)	mild ALVDD (n = 169)	HFpEF (n = 77)	
Age (years)	54 (48-61)	66 (58-71)	73 (68-77)	< 0.001*
BMI (kg/m^2^)	25.5 (24.1-29.1)	27.8 (25.6-32.3)	27.5 (25.7-32.0)	0.001*
Waist circumference (cm)	98 (86-107)	102 (94-114)	102 (98-111)	0.002*
Hip circumference(cm)	98 (94-103)	103 (96-111)	105 (98-114)	0.003*
Systolic BP (mmHg)	125 (110-136)	134 (127-140)	136 (130-140)	0.001*
Diastolic BP (mmHg)	80 (70-80)	80 (76-84)	80 (72-84)	0.12
Resting HR (beats/min)	70 (68-76)	72 (69-76)	70 (65-76)	0.51
CAD, n (%)	21 (38,2)	99 (58,6)	49 (63,6)	0.009*
CABG, n (%)	1 (2.0)	5 (3.0)	9 (11.7)	0.007*
PCI, n (%)	15 (27.3)	75 (44.4)	33 (42.9)	0.074
History of MI, n (%)	8 (14.5)	36 (21.3)	17 (22.1)	0.501
History of stroke, n (%)	1 (2.0)	5 (3.0)	3 (3.9)	0.824
Cardiovascular risk factors				
Treated hypertension, n (%)	38 (69.1)	148 (88.1)	73 (96.1)	< 0.001*
Diabetes mellitus, n (%)	4 (7.3)	45 (26.6)	22 (28,6)	< 0.001*
Medication				
ACE inhibitor, n (%)	26 (47.3)	110 (65.1)	42 (54.5)	0.042*
AT1 receptor blocker, n (%)	6 (10.9)	17 (10.1)	23 (29.9)	< 0.001*
Diuretics, n (%)	8 (14.5)	45 (26.6)	36 (49.8)	< 0.001*
Ca^2^ blocker, n (%)	6 (10.9)	23 (13.6)	21 (27.3)	0.013*
Β-blocker, n (%)	28 (50.9)	103 (60.9)	57 (74.0)	0.021*

* statistically significant (p < 0.05). BMI: body mass index; BP: blood pressure; HR: heart rate; CAD: coronary artery disease; CABG: coronary artery bypass graft; PCI: percutaneous coronary intervention; MI: myocardial infarction; DF: diastolic function; ALVDD: left ventricular diastolic dysfunction; HFpEF: heart failure with preserved ejection fraction; NS: non-significant. The Mann-Whitney U-test was used to analyze differences between the medians of two groups and the Kruskal-Wallis test to test the equality of medians among more than two distinct groups.

### Endostatin and diastolic function

**Figure 1:**
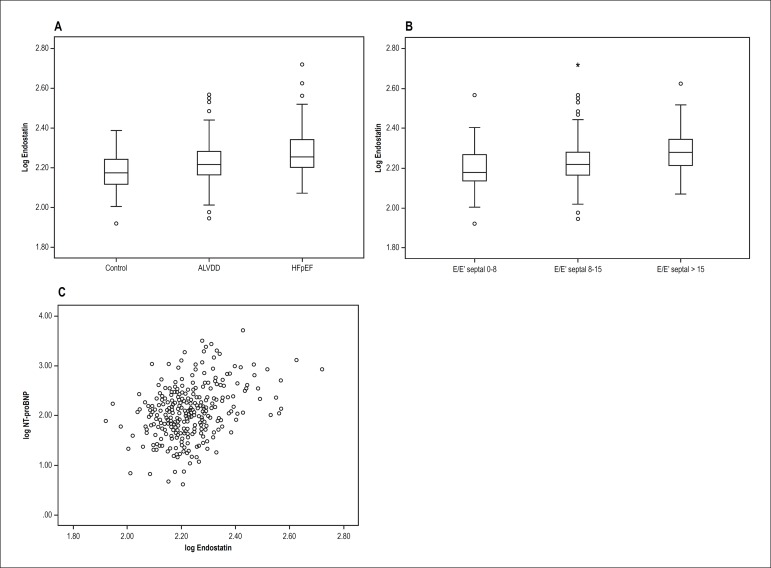
(A) The boxplot graphics show serum Endostatin levels for the ALVDD, HFpEF patients and the control group. (B)The correlation between Endostatin levels and the E/E' ratio as surrogate for increased left ventricular filling pressures. (C) The logarithmic dot blot displays the correlation of Endostatin serum levels with NT-proBNP.

Table 2 summarizes the laboratory data and echocardiographic function parameter stratified by the study groups HFpEF vs. ALVDD vs. controls. Levels of Endostatin were 179.0 [159-220] ng/mL in HFpEF, 163.8 [145.4-191.3] ng/mL in ALVDD and 149 [130.6 - 176.9] ng/mL in the control group, respectively (Figure 1A). Serum levels of Endostatin were significantly higher in patients with HFpEF (p < 0.001) and mild ALVDD (p = 0.001; Table 2) compared to individuals from the control group. Furthermore, Endostatin serum concentration was elevated in patients with mild ALVDD compared to asymptomatic controls with normal diastolic and systolic function (p = 0,004). In addition, there was a significant association between increasing Endostatin quartiles and higher NT-pro-BNP levels. No clinically relevant differences were observed in the clinical routine laboratory assessments. In multivariable analysis included the covariates Endostatin, age, gender, diabetes, hypertension, coronary artery disease and body mass index, age (p < 0.001) and Endostatin (p = 0.008 were independently associated with HFpEF

**Table 2:** Laboratory data and echocardiographic parameters. (25-75interquartile range) or absolute numbers and percentage (%) X^2^ test was used as appropriate

Clinical variables	Studied patient groups			p value
	Control (n = 55)	mild ALVDD (n = 169)	HFpEF (n = 77)	
**Biomarker**				
Endostatin (ng/ml)	149.1 (130.6-176.9)	163.8 (145.4-191.3)	179.0 (159-220)	< 0.001*
NT-pro-BNP (pg/ml)	90.1 (45.8-129.2)	86.7 (43.7-170.7)	343.6 (151.7-703.4)	< 0.001*
**Routine parameter**				
Total cholesterol (mg/dl)	189 (163-228)	193 (171-221)	191 (170-210)	NS
LDL-cholesterol (mg/dl)	107 (89-135)	109 (89-135)	109 (86-129)	NS
HDL-cholesterol (mg/dl)	53 (46-64)	50 (38-62)	48 (41-61)	NS
Triclyceride (mg/dl)	119 (83-185)	142 (100-206)	131 (104-189)	NS
Lp (a) (mg/dl)	8 (5-27)	18 (6-39)	15 (6-52)	NS
TSH (mU/l)	1.20 (0.94-2.09)	1.42 (0.824-2.08)	1.315 (0.80-1.90)	NS
Creatinine (mg/dl)	0.8 (0.7-0.9)	0.9 (0.7-0.9)	0.9 (0.75-1.10)	NS
hsCRP	0.1 (0.1-0.3)	0.3 (0.1-0.6)	0.3 (0.2-0.69)	0.005*
Glucose	89 (84-97)	100 (91-111)	97 (89-103)	0.020*
Hb (mg/dl)	14.3 (13.3-15.1)	14.1 (13.2-15.0)	13.6 (12.5-14.5)	0.004*
CK (U/l)	76 (58-105)	78 (60-114)	72 (55-104)	NS
SGOT (U/l)	25 (21-31)	25 (21-31)	26 (21-32)	NS
**LV geometry**				
IVS (mm)	10 (9-11)	12 (10-13)	12 (11-14)	< 0.001*
PLW (mm)	10 (9-11)	12 (10-13)	12 (11-14)	< 0.001*
LVEDD(mm)	44 (42-47)	44 (39-48)	45 (41-50)	NS
LVESD (mm)	30 (28-34)	29 (25-34)	31 (27-36)	NS
LVMi (g/m^2^)	72 (62-84)	81 (67-102)	91 (77-119)	< 0.001*
**Systolic function**				
EF (%)	68 (62-72)	67 (61-71)	67 (63-73)	NS
S_max_ (cm/s)	7.2 (6.3-8.0)	6.3 (5.7-7.5)	6.1 (5.4-6.7)	< 0.001*
**Diastolic function**				
LA-Index (ml/m^2^)	25.4 (21.8-28.7)	29.8 (25.7-33.3)	39.3 (36.7-49.1)	< 0.001*
E (cm/s)	60 (60-80)	60 (50-70)	80 (70-90)	< 0.001*
A (cm/s)	60 (50-70)	80 (70-90)	80 (70-90)	< 0.001*
E/A ratio	1.14 (0.68-1.25)	0.75 (0.67-0.86)	1.11 (0.85-1.25)	< 0.001*
E'septal (cm/s)	8.4 (7.3-9.4)	5.9 (5.2-6.8)	5.4 (4.6-6.3)	< 0.001*
E'lateral (cm/s)	10.7 (9.5-13.0)	8.2 (6.9-9.5)	6.9 (5.6-8.4)	< 0.001*
Average E'(cm/s)	9.8 (8.6-11.0)	7.2 (6.1-8.1)	6.2 (5.2-7.2)	< 0.001*
E/E'septal ratio	8.0 (6.9-9.0)	10.2 (8.3-11.9)	15.1 (12.5-17.1)	< 0.001*
E/E'average ratio	7.0 (6.0-7.7)	8.4 (6.8-10.1)	13.3 (11.1-14.8)	< 0.001*

* statistically significant (p < 0.05); NT-proBNP: N-terminal fragment of the prohormone B-type natriuretic peptide; LDL: low density lipoprotein; HDL: high density lipoprotein; Lp (a): lipoprotein (a); TSH: thyroid stimulating hormone; hsCRP: high sensitive C-reactive protein; Hb: hemoglobin; CK: creatinkinase; SGOT: serum glutamic oxaloacetic transaminase; IVS: interventricular septum; PLW: posterior lateral wall; LVEDD: left ventricular end-diastolic diameter; LVESD: left ventricular end-systolic diameter; EF: ejection fraction; LA: left atrial; E: early diastolic transmitral velocity; A: late diastolic transmitral velocity; E': early diastolic tissue Doppler velocity; DF: diastolic function; LVDD: left ventricular diastolic dysfunction; HFpEF: heart failure with preserved ejection fraction; NS: non-significant. The Mann‑Whitney U-test was used to analyze differences between the medians of two groups and the Kruskal-Wallis test to test the equality of medians among more than two distinct groups.

### Association of Endostatin levels with cardiac structure and function

Increasing quartiles of Endostatin were significantly associated with structural changes of the heart like the extent of LV- hypertrophy and left atrial enlargement, reflecting adverse cardiac remodeling. Moreover, increasing quartiles of Endostatin were significantly associated with worsening diastolic function measured by tissue Doppler imaging (E', E/E') (table 3). Thus, patients within the highest quartiles of Endostatin serum levels showed more advanced cardiac remodeling (LV hypertrophy and left atrial enlargement) as well as more severe diastolic function abnormalities reflecting increasing left ventricular filling pressures (Figure 1B). Consistently, there was a significant positive moderate correlation between Endostatin and NT-proBNP levels (r = 0.32, p < 0.001; Figure 1C).

**Table 3:** Echocardiographic parameters stratified according to serum Endostatin quartiles. Values are median (interquartile range) or n (%). X^2^ test was used as appropriate

Parameter	Endostatin 1^rd^ quartile	Endostatin 2^nd^ quartile	Endostatin 3^rd^ quartile	Endostatin 4^th^ quartile	p value
**LV geometry**					
IVS (mm)	11 (9-12)	12 (11-13)	12 (11-13)	12 10-14)	0.032^*^
PLW (mm)	11 (10-13)	11 (10-13)	12 (10-13)	12 (10-14)	NS
LVEDD (mm)	44 (41-47)	43 (40-47)	47 (40-49)	45 (40-48)	NS
LEVSD (mm)	30 (27-34)	29 (26-32)	31 (27-35)	29 (24-37)	NS
LVMi (g/m²)	76.4 (61.6-100.4)	74.2 (66.2-97.8)	87.8 (72.9-100.3)	94.5 (70.8-117.1)	0.024*
**Systolic function**					
Ejection fraction (%)	65 (60-70)	68 (63-72)	67 (61-71)	69 (63-74)	0.029*
S_max_ (cm/s)	6.6 (5.8-7.7)	6.6 (5.8-7.7)	6.4 (5.6-7.2)	6.1 (5.3-7.0)	0.005*
**Diastolic function**					
LA-Index (ml/m^2^)	28.6 (23.8-35.3)	31.2 (25.7-35.2)	29.9 (25.7-36.9)	33.4 (27.9-38.8)	0.023*
E (cm/s)	60 (50-75)	60 (50-70)	70 (50-80)	70 (60-80)	NS
A (cm/s)	70 (60-80)	70 (60-80)	80 (70-90)	80 (70-95)	< 0.001*
E/A ratio	0.9 (0.7-1.2)	0.9 (0.7-1.1)	0.8 (0.7-1.0)	0.8 (0.7-1.1)	NS
E'septal (cm/s)	6.9 (5.6-8.0)	6.0 (5.3-7.3)	6.3 (5.3-7.3)	5.6 (4.9-6.2)	< 0.001*
E'lateral (cm/s)	9.1 (7.1-10.7)	8.6 (7.0-10.2)	8.3 (7.0-10.2)	7.5 (6.3-8.9)	0.001*
Average E'	7.9 (6.8-9.3)	7.4 (6.3-8.5)	7.6 (6.2-8.3)	6.4 (5.5-7.5	< 0.001*
E/E'septal ratio	8.8 (7.5-11.4)	10.3 (8.3-12.5)	10.8 (8.3-13.0)	12.1 (9.8-15.8)	< 0.001*
E/E'average ratio	7.5 (6.5-9.8)	8.5 (7.1—10.5)	8.9 (7.1-11.5)	10.5 (8.4-13.1)	< 0.001*
**Laboratory**					
NT-pro-BNP (pg/ml)	81.40 (45.1-137.3)	93.25 (43.70-211.6)	104.6 (52.8-179.7)	218.2 (100.35-516.15)	< 0.001*

* statistically significant (p < 0.05). IVS: interventricular septum; PLW: posterior lateral wall; LVEDD: left ventricular end-diastolic diameter; LVESD: left ventricular end‑systolic diameter; EF: ejection fraction; LA: left atrial; E: early diastolic transmitral velocity; A: late diastolic transmitral velocity; E': early diastolic tissue Doppler velocity; NT‑proBNP: N-terminal fragment of the prohormone B-type natriuretic peptide; DF: diastolic function; LVDD: left ventricular diastolic dysfunction; NS: non‑significant, HFpEF, heart failure with preserved ejection fraction. The Mann-Whitney U-test was used to analyze differences between the medians of two groups and the Kruskal‑Wallis test to test the equality of medians among more than two distinct groups.

## Discussion

We hypothesized that circulating Endostatin levels are altered in patients with ALVDD and HFpEF. Furthermore, elevated levels are associated with the presence and severity of diastolic function abnormalities. To verify the hypothesis we performed a clinical observational study including 301 patients, which were assigned based on their echocardiographic characteristics to three different groups. To our knowledge, this is the first published report linking increased circulating Endostatin levels to the presence and severity of diastolic function abnormalities and HFpEF in a well phenotyped cohort of patients with normal systolic function. In the present study, Endostatin showed a graded increase from controls over ALVDD to HFpEF. Furthermore, higher Endostatin levels were significantly associated with established markers of structural cardiac abnormalities including the LAVi and increased LV mass as well as functional abnormalities like E/E' ratio. Particularly, an increased LAVi without concomitant mitral valve disease reflects a chronic remodeling process typical for HFpEF.^25^ Consistently, we found that elevated Endostatin levels were associated with elevated NT-proBNP levels, a well-recognized prognostic marker and indicator of elevated ventricular filling pressures among patients, independent from LVEF.^26^

Endostatin, a 20-kDa proteolytic fragment from the C-terminal domain of collagen XVIII, was shown to have an inhibitory effect on tumor growth working as an antiangiogenic growth factor.^27^ Endostatin plays a role in the local balance of angiogenesis and shows potent anti-angiogenic activity by inhibiting proliferation and migration of endothelial cells in addition to inducing endothelial cell apoptosis.^27^ Endostatin is produced by the proteolytic cleavage of the C-terminal domain of collagen XVIII, a component of the extracellular matrix. The precise mechanism of conversion from collagen XVIII to Endostatin has not yet been fully elucidated.^28,29^ Recent studies of patients with coronary artery disease (CAD) demonstrate that Endostatin protein levels correlate significantly with reduced angiogenesis and poorly developed cardiac collateral vasculature.^18,30^

The results from our study fit well to the pathophysiological model used to explain the development of HFpEF. In general, HFpEF is a complex disease involving an interplay of various factors. There is the hypothesis that a failure of oxygen delivery to the cardiomycytes triggers a pro-angiogenic response in patients suffering from heart failure.^31-33^ Nonetheless, angiogenic and antiangiogenic growth factors often co-exist in tissues with angiogenesis.^34^ Thus, the status of endothelial cells and endothelial function is determined by a balance between these positive and negative factors on angiogenesis, and the balance may in inappropriate shifted towards antiangiogenic factors in patients with HF. It was shown that the role of microvascular dysfunction and microvascular inflammation is especial for patients with the diagnosis of HFpEF.^5,35,36^ A new pathophysiologic model presented by Redfield et al.^8^ points from pro-inflammatory coexisting conditions to systemic endothelial inflammation and impaired oxygen delivery.^8^ Global ventricular performance is highly dependent on oxygen supply and thus myocardial perfusion, and an essential component of myocardial perfusion during ventricular hypertrophy is the myocyte-microvascular balance and the myocyte/capillary ratio. In cardiac autopsy specimens, it has recently been shown that microvascular rarefaction is a downstream phenomenon in HFpEF.^37^ Furthermore, Kitzman et al.^38^ has demonstrated that HFpEF patients display significant abnormalities in the skeletal muscle as well as an abnormal capillary-to-fiber ratio, probably building the basis for severe exercise intolerance in HFpEF patients.^38^ In addition, Gouya et al.^19^ have shown in a relatively small HFrEF study population that high levels of serum Endostatin were associated with all-cause mortality and concluded that the effect of increased angiogenesis is HF may be blunted by an overspill of anti-angiogenic factors such as Endostatin.^19^ Thus, we hypothesize that similar pathophysiological concepts may be involved in patients with HFpEF, were a high proportion of patients has a coincidence of coronary artery disease and diabetes, both damaging the endothelial structure.^39^ This was also true for our patient population as shown in table 1. Endostatin could be moderator of the microvascular effects seen in these patients.^40^

Several limitations of this study must be acknowledged. The observational nature of the present study prohibits definitive determination of cause and effect relationships. Second, the present study was a single-center experience with a relatively small number of subjects. Third, longitudinal follow-up data were not available to test associations between the Endostatin serum levels and clinical outcomes. Moreover, we enrolled consecutive patients referred for elective coronary angiography and echocardiography, which may not represent a general population cohort without evidence or suspicious for cardiovascular diseases.

Further studies should include more patients from a broader population and capture longitudinal data including information about hospitalization and mortality.

## Conclusion

In this exploratory hypothesis-generating study, we provide first evidence that Endostatin correlates with the presence and severity of diastolic dysfunction and HFpEF and may become a novel biomarker for the diagnosis and stratification of HFpEF. Increased Endostatin levels may reflect deterioration of diastolic function caused by adverse remodeling. Further prospective studies are needed to determine the causal relationship as well as the diagnostic and prognostic value of Endostatin in HFpEF and the potential role as a therapeutic target.
